# Molecular Evolution of a Viral Non-Coding Sequence under the Selective Pressure of amiRNA-Mediated Silencing

**DOI:** 10.1371/journal.ppat.1000312

**Published:** 2009-02-27

**Authors:** Shih-Shun Lin, Hui-Wen Wu, Santiago F. Elena, Kuan-Chun Chen, Qi-Wen Niu, Shyi-Dong Yeh, Chin-Chih Chen, Nam-Hai Chua

**Affiliations:** 1 Laboratory of Plant Molecular Biology, Rockefeller University, New York, New York, United States of America; 2 Instituto de Biología Molecular y Celular de Plantas (CSIC-UPV), Valencia, Spain; 3 Department of Plant Pathology, National Chung Hsing University, Taichung, Taiwan; 4 Division of Plant Pathology, Agricultural Research Institute, Wu-Feng, Taichung, Taiwan; University of California Riverside, United States of America

## Abstract

Plant microRNAs (miRNA) guide cleavage of target mRNAs by DICER-like proteins, thereby reducing mRNA abundance. Native precursor miRNAs can be redesigned to target RNAs of interest, and one application of such artificial microRNA (amiRNA) technology is to generate plants resistant to pathogenic viruses. Transgenic *Arabidopsis* plants expressing amiRNAs designed to target the genome of two unrelated viruses were resistant, in a highly specific manner, to the appropriate virus. Here, we pursued two different goals. First, we confirmed that the 21-nt target site of viral RNAs is both necessary and sufficient for resistance. Second, we studied the evolutionary stability of amiRNA-mediated resistance against a genetically plastic RNA virus, TuMV. To dissociate selective pressures acting upon protein function from those acting at the RNA level, we constructed a chimeric TuMV harboring a 21-nt, amiRNA target site in a non-essential region. In the first set of experiments designed to assess the likelihood of resistance breakdown, we explored the effect of single nucleotide mutation within the target 21-nt on the ability of mutant viruses to successfully infect amiRNA-expressing plants. We found non-equivalency of the target nucleotides, which can be divided into three categories depending on their impact in virus pathogenicity. In the second set of experiments, we investigated the evolution of the virus mutants in amiRNA-expressing plants. The most common outcome was the deletion of the target. However, when the 21-nt target was retained, viruses accumulated additional substitutions on it, further reducing the binding/cleavage ability of the amiRNA. The pattern of substitutions within the viral target was largely dominated by G to A and C to U transitions.

## Author Summary

RNA viruses are well-known for their tremendous capacity to evolve, a characteristic that threatens the development of effective antiviral strategies. A new antiviral strategy was recently proposed to control plant RNA viruses that relied on the expression in plants of artificial microRNAs (amiRNAs) targeting short sequences of 21-nt in the viral genome. Here, we have evaluated the likelihood that changes in the 21-nt target sequence would result in resistance breakdown. We found that changes at different sites in the target had different consequences on the ability of the virus to evade amiRNA surveillance. Then, we evolved viruses with a single substitution within the target under the selective pressure imposed by amiRNAs but without any selective pressure at the protein level. We found extra mutations accumulated in the target that further reduced base pairing with the amiRNA. These results showed that when allowed to replicate, RNA viruses would readily generate genetic variability that would facilitate evasion from the engineered miRNA-mediated virus resistance.

## Introduction

Plant miRNAs regulate the abundance of target mRNAs by guiding their cleavage at the sequence complementary region. Previous reports have shown that changes of several nucleotides within a miRNA 21-nt sequence do not affect its biogenesis and maturation [1],[2]. This finding raises the possibility to redesign the miRNA sequence to target specific transcripts, originally not under miRNA control. Such artificial miRNAs have been produced in dicotyledonous [3]–[5] and monocotyledonous plants [6] using different pre-miRNAs as backbones. We have successfully demonstrated that redesigned artificial miRNAs (amiRNAs) are biologically active and can be used to confer specific virus resistance in transgenic plants [4]. The pre-miR159a precursor was used to generate two amiRNA^159^s (amiR^159^-P69 and amiR^159^-HC-Pro) with sequence complementary to the RNA genome of two plant viruses, *Turnip yellow mosaic virus* (TYMV) and *Turnip mosaic virus* (TuMV), respectively. The amiR-P69 was designed to target sequences encoding the P69 suppressor of TYMV whilst amiR^159^-HC-Pro would target sequences for the HC-Pro silencing suppressor of TuMV. Transgenic lines carrying both 35S-pre-amiR^159^-P69 and 35S-pre-amiR^159^-HC-Pro transgenes can express the appropriate amiRNA at high levels and showed specific resistance to either TYMV and TuMV, depending on the expression of the cognate amiRNA [4]. Specific resistance to TuMV was also seen with plants expressing amiR^159^-TuCP directed against the TuMV coat protein (CP) gene [4].

In animal systems, RNA interference (RNAi), a gene-silencing mechanism similar to that of miRNA, has been used in clinical trials as antiviral therapeutics to inhibit replication of several human pathogenic viruses (reviewed in [7],[8]). As demonstrated for HIV-1, a major problem of RNAi-mediated antiviral therapies is the emergence of resistant virus variants, which differ from the wild type virus by having fixed point mutations in the target sequence leading to imperfect matching; these mutant viruses are not properly processed by the enzymatic silencing machinery [9]–[13]. Some mismatches within the target sequence are tolerated by the RNAi machinery whereas other mismatches, such as those in the central region (position 9 to 11) of the target sequence, compromise RNAi-guided antiviral therapies [14],[15]. However, all these studies suffer from the drawback of having a superimposition of two different selective forces: on the one hand, purifying selection acts at the protein level (i.e., the necessity of maintaining a functional protein) and, on the other hand, diversifying selection acts at the RNA sequence level favoring mutant genomes capable of evading RNA silencing.

Mallory et al (2004) have used an *in vitro* wheat germ system to assay for critical positions within a miRNA target site needed for efficient plant mRNA cleavage [16]. Analysis of scanning mutants revealed that mismatches at the center and the 3′ end of the miRNA are more tolerated compared to mismatches at the 5′ region [16]. Recently, the molecular mechanism of RISC-mediated RNA cleavage has been investigated by *in vitro* reconstitution assays using human RISC [17]–[19]. It was found that the accessibility of RNA target site correlates directly with the RNA cleavage efficiency, indicating that RISC is unable to unfold structured RNA. In the course of target recognition, RISC transiently contacts single-stranded RNA nonspecifically and promotes siRNA-target RNA annealing. Furthermore, the 5′ portion of the siRNA within RISC creates a thermodynamic threshold that determines the stable association of RISC and the target RNA. Furthermore, in addition to this clear position-effect, overall desestabilization of the double strand structure has little effect on RNAi activity until an energy threshold is reached, beyond of which a negative correlation exist between stability and RNAi-mediated inhibition [15].

Here, we first investigated whether the 21-nt of an amiRNA target site is both necessary and sufficient for amiRNA-mediated specific resistance. Second, we were interested in identifying critical positions within the target site for this resistance. Third, we have explored the patterns of sequence polymorphism of viral sequences that evolve under the only selective pressure of amiRNA-mediated silencing. To address these issues, we established a heterologous-virus resistance system using a TuMV-GFP viral vector to carry a non-essential 21-nt sequence of the *P69* gene targeted by amiR^159^-P69. This heterologous-virus system allows us to modify any nucleotide within the 21-nt target site without altering virus coding sequences and thus without affecting replication and activity. In other words, this heterologous system allows separating the selective pressure imposed by protein functionality from the selective pressure imposed at the sequence level by RNA silencing. The 21 scanning mutant viruses were inoculated on amiR^159^-P69 plants and the proportion of transgenic plants that became infected was used to determine the importance of the mutated nucleotide position within the amiRNA target site.

## Results

### Construction of the 21-nt target site in TuMV

**Figure 1:**
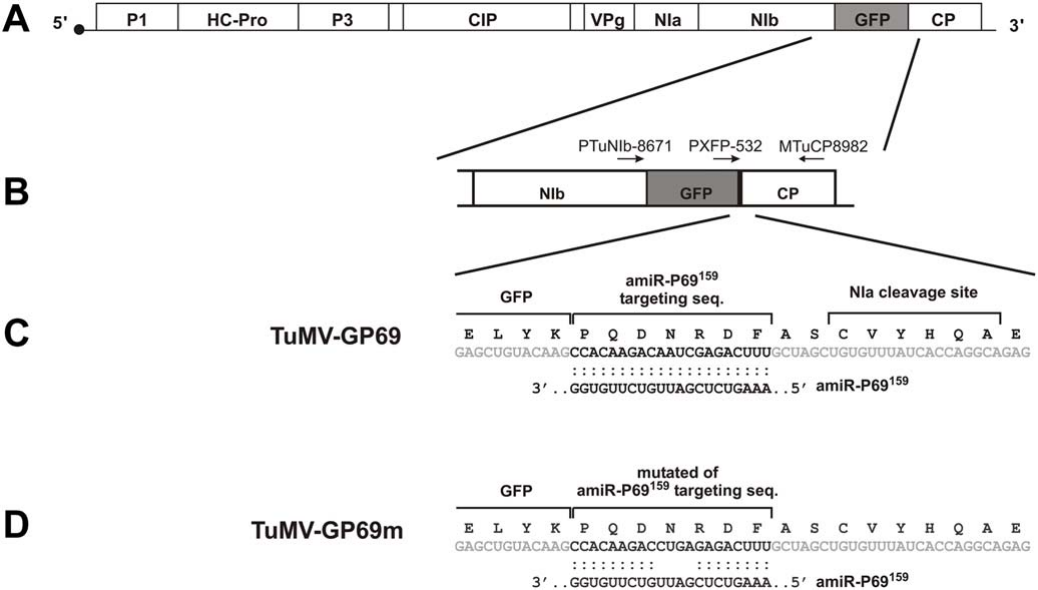
Schematic representations of infectious clones of chimeric *Turnip mosaic virus* (TuMV). (A) Schematic representation of TuMV-GFP infectious clone carrying a GFP gene inserted between the *NIb* and *CP* genes. (B) Arrows represent the positions and orientations of primers used for RT-PCR. The primer sets PTuNIb-8671/MTuCP-8982 and PXFP-532/MTuCP-8982 were used to amplify the NIb-CP and GFP-CP regions, respectively. (C,D) Schematic diagrams showing the 21-nt sequence of P69 and P69m in the chimeric viruses TuMV-GP69 and TuMV-GP69m, respectively. Predicted base pairing of the 21-nt target RNA sequence (top strand) and amiR^159^-P69 (bottom strand) are shown below the amino acid sequence of the TuMV-GFP poly-protein.

We have previously demonstrated that a 21-nt amiRNA, with sequence complementary to a viral sequence, can mediate cleavage of target viral RNA and confer resistance on transgenic plants [4]. However, it was not known whether the 21-nt viral target site, complementary to the amiRNA sequence, was sufficient for specific resistance. To this end, we constructed a *green fluorescence protein* (*GFP*) gene carrying a 21-nt sequence (5′-CCACAAGACAAUCGAGACUUU-3′) of the TYMV *P69* gene at its 3′-end and inserted the *GFP-P69_21nt_* fusion gene in between the *NIb* and *CP* genes to generate a TuMV-GP69 chimeric virus (Fig. 1). As a control, we mutated 4 nts (position 9 to 12 from the 3′-end; underline) of the target 21-nt sequence (5′-CCACAAGACCUGAGAGACUUU-3′) to give *GFP-P69_21nt_m*, which was inserted in the same position of the viral genome to generate the TuMV-GP69m chimeric virus (Figure 1D).

The presence of two TuMV NIa protease cleavage sites (CVYHQA) at both the N- and C-termini of the GFP-P69_21nt_ fusion protein allows the release of GFP plus a 7 amino acid C-terminal extension (PQDNRDF) from the TuMV-GFP viral polyprotein (Figure 1C and 1D). Virus infection can be easily confirmed and followed by monitoring GFP signals from infected tissues.

### amiRNA-mediated specific resistance to heterologous virus

**Figure 2:**
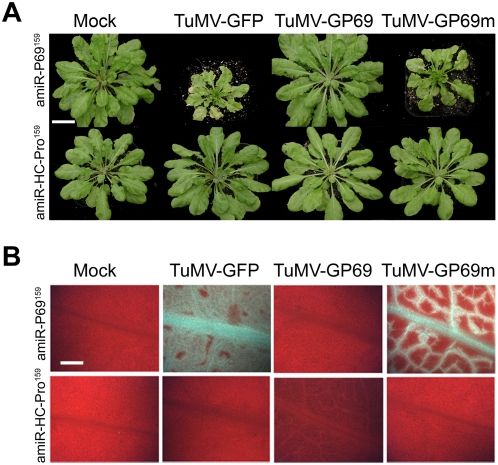
A 21-nt sequence targeted by amiRNA is necessary and sufficient to confer virus resistance. (A) amiR^159^-P69 and amiR^159^-HC-Pro transgenic *Arabidopsis* plants were mock-inoculated or inoculated with TuMV-GFP, TuMV-GP69, or TuMV-GP69m. As controls, the same transgenic lines were inoculated with TuMV-GFP. Photographs were taken at 12 dpi. Bar = 0.5 cm. (B) GFP fluorescence of systemic leaves of plants infected with chimeric viruses. Leaves were examined by fluorescence microscopy. Leaves of sensitive amiR^159^-P69 plants displayed green fluorescence due to replication of GFP-virus, whereas no green fluorescence was detected in leaves of resistant amiR^159^-HC-Pro or amiR^159^-P69 plants. Bar = 2 cm.

We have previously shown that transgenic *Arabidopsis thaliana* plants expressing amiR-P69 can specifically target the *P69* gene of TYMV and displayed specific resistance to TYMV [4] although these plants remained susceptible to heterologous virus (TuMV-GFP) infection (Figure 2A, top second panel). Figure 2 shows that insertion of the 21-nt sequence of the TYMV *P69* gene into TuMV-GFP, which was targeted by amiR^159^-P69, rendered these amiR^159^-P69 plants resistant to TuMV-GP69 (Figure 2A, top third panel). Control experiments showed that the amiR^159^-P69 plants remained sensitive to TuMV-GP69m (Figure 2A, top fourth panel), which carried 4 mutations in the central region of the 21-nt site of the P69 gene. Systemic leaves of amiR^159^-P69 plants displayed GFP fluorescence when inoculated with TuMV-GFP or TuMV-GP69m (Figure 2B, top second and fourth panels), but no GFP signal was detected upon TuMV-GP69 inoculations (Figure 2B, top third panel).

Plants expressing amiR^159^-HC-Pro were resistant to chimeric TuMV-GFP, TuMV-GP69 and TuMV-GP69m and no GFP was detected on systemic leaves of inoculated plants (Figure 2B, bottom panels). These results were expected since all these 3 chimeric viruses contained the HC-Pro gene targeted by amiR^159^-HC-Pro [4].

### Expression of amiR^159^-P69 in *Nicotiana benthamiana* confers resistance to TuMV-GP69

**Figure 3:**
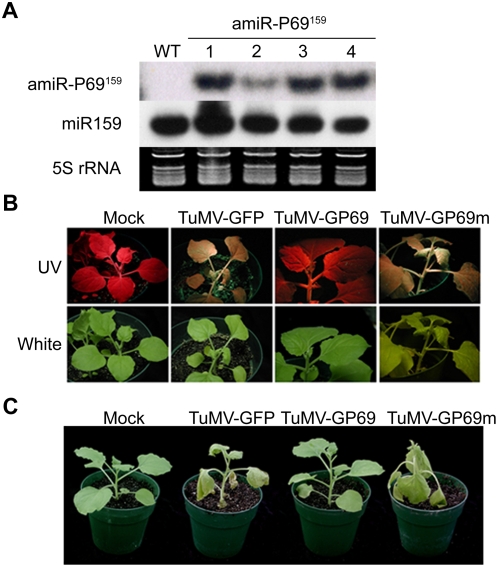
Transgenic *N. benthamiana* plants expressing amiR^159^-P69 are resistant to infection by chimeric TuMV virus. (A) amiR^159^- P69 expression levels of transgenic *N. benthamiana* plants carrying 35S-pre-amiR^159^-P69. Four independent lines (# 1, 2, 3, and 4) were analyzed. 5S rRNAs were used as a loading control. (B) Early infection of chimeric TuMV viruses monitored by UV excitation. (C) amiR^159^-P69 *N. benthamiana* plants were resistant to TuMV-GP69 but susceptible to TuMV-GP69m or TuMV-GFP.

Next, we established the heterologous virus resistance system in *N. benthamiana* and tested amiRNA-mediated resistance efficiency. Figure 3A shows amiR^159^-P69 expression levels in 4 independent transgenic *N. benthamiana* lines (#1, 2, 3, and 4). Progeny plants of these lines were challenged with TuMV-GFP, TuMV-GP69 or TuMV-GP69m.

The GFP signal produced by infection with TuMV-GFP or TuMV-GP69m can be detected at 4 dpi (early stage of symptom development) (Figure 3B). As expected, at 7 dpi, transgenic *N. benthamiana* plants expressing amiR^159^-P69 were resistant to TuMV-GP69 but susceptible to TuMV-GFP and TuMV-GP69m (Figure 3C). Plants that were sensitive to virus infection showed severe wilting symptoms (Figure 3C). These results, which were very similar to those obtained with *A. thaliana* transgenic plants, provided further confirmation that the targeted 21-nt site is necessary and sufficient for specific amiRNA-mediated specific resistance. In addition, the results also suggested that pre-amiR^159^-P69, which is a modified form of the *Arabidopsis* miR^159^ precursor, can be processed by *N. benthamiana* plants to produce functional amiR^159^-P69 to confer virus resistance.

### Scanning mutagenesis of the 21-nt sequence targeted by amiR^159^-P69

As the 4 nt mutation on the central positions of the target sequence (position 8 to 12) compromised specific resistance, we decided to further investigate nucleotide positions within this 21-nt sequence that are critical for amiRNA-mediated resistance. Note that the sequence can be systematically altered without affecting essential viral gene functions because the amiR^159^-P69 target sequence is non-essential to the TuMV-GP69 chimeric virus.

**Figure 4:**
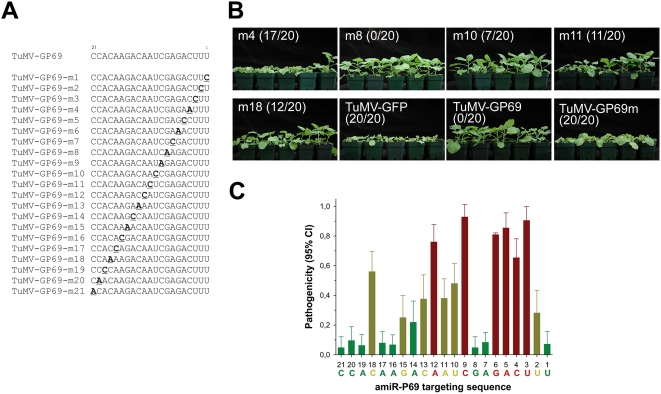
Scanning mutagenesis of the amiR^159^-P69 target site on TuMV-GP69 chimeric virus. (A) A schematic representation of the 21 scanning mutants with substitution of single nucleotide within the 21-nt sequence targeted by amiR^159^-P69. (B) Representative amiR^159^-P69 *N. benthamiana* plants displaying different degree of breakdown when inoculated with the scanning mutants. The ratio in each panel indicates the number of susceptible amiR^159^-P69 plants amongst 20 plants challenged. (C) A summary of critical positions within the amiR^159^-P69 target site. The 21-nt RNA sequence is shown on the *x*-axis. Numbers below the sequence indicate the positions of amiR^159^-P69 starting from the 5′ end. The degree of resistance breakdown was represented as the percent of inoculated plants with viral disease symptoms. Red bars represent critical positions for resistance; yellow bars represent positions of moderate importance; green bars represent positions of minimal influence in resistance-breakdown.

Accordingly, we generated a series of mutants by making all possible synonymous scanning substitutions within the 21-nt sequence of *P69* in the background of TuMV-GP69 (Figure 4A). Each A of the *P69* viral sequence that pairs to a U of amiR^159^-P69 was changed to a C to create a C∶U mismatch; each C and G of the viral sequence was changed to an A to create A∶G and A∶C mismatches; and each U of the viral sequence was changed to a C to create C∶A mismatches.

A total of 21 mutant viruses with single nt substitution from the 1^st^ to the 21^st^ position of the target site were used to challenge non-transgenic WT and amiR^159^-P69 *N. benthamiana* plants. The proportion of inoculated amiR^159^-P69 plants that showed visible symptoms after inoculation, i.e. pathogenicity, was used as a measure of the importance of the mutated nucleotide within the 21-nt target site in amiR^159^-P69–mediated specific resistance.

We used TuMV-GFP, TuMV-GP69, and TuMV-GP69m as controls. Whereas WT plants were susceptible to TuMV-GP69 no symptoms developed in amiR^159^-P69 plants even at 10 dpi (Table 1, and Figure 4B bottom third panel). By contrast, TuMV-GFP and TuMV-GP69m elicited 100% infection on WT as well as amiR^159^-P69 transgenic tobacco plants, and these infected plants displayed symptoms at 5 dpi (Table 1, and Figure 4B bottom second and fourth panel).

**Table 1:** Pathogenicity of -nt substitution of chimeric TuMV-GFP viruses on amiR^159^-P69 plants.

	Number of Infected/Inoculated Plants	Days of Delay in Symptoms Relative to TuMV-GFP	Pathogenicity (±95 CI) (%)
TuMV-GFP	58/58	0	98.33±3.12
TuMV-GP69	0/58	Never	1.67±3.12
TuMV-GP69m	48/48	0	98.00±3.72
TuMV-GP69-			
m1	2/40	4	7.14±8.45
m2	8/30	2	28.13±15.34*
m3	37/40	1	90.48±9.34*
m4	33/50	1	65.38±12.75*
m5	40/46	1	85.42±10.15*
m6	33/40	1	80.95±11.92*
m7	5/70	3	8.33±6.65*
m8	1/40	2	4.76±7.38
m9	38/40	2	92.86±8.45*
m10	23/48	1	48.00±13.60*
m11	18/48	1	38.00±13.24*
m12	37/48	1	76.00±11.78*
m13	11/30	2	37.50±16.37*
m14	6/30	3	21.88±14.27*
m15	7/30	2	25.00±14.84*
m16	3/58	2	6.67±6.74
m17	3/49	3	7.84±7.84
m18	27/48	2	56.00±13.52*
m19	2/46	2	6.25±7.47
m20	3/40	1	9.52±9.34*
m21	1/40	2	4.76±7.38

*: Pathogenicity values significantly greater than zero.

WT plants were 100% susceptible to all 21 scanning mutant viruses and symptoms appeared at 5 dpi (data not shown), indicating that the single nt substitutions on the target 21-nt sequence did not affect mutant virus replication nor movement. On the other hand, these mutant viruses showed variable pathogenicity after inoculation on amiR^159^-P69 plants (Table 1, and Fig 4B top panels & bottom first panel). Fifteen mutants showed pathogenicity values that were significantly greater than zero (Table 1). For these pathogenic mutants, the percentage of infected plants ranged from 8.33% (m7) to 92.86% (m9). Mutants were classified according to their pathogenicity using a 2-step cluster analysis. The minimum number of clusters into which the mutants can be significantly partitioned was three (Bayesian weight 98.44%; Kruskal-Wallis test: *H* = 17.739, 2 d.f., *P*<0.001). Figure 4C assigns mutants to the different clusters. The first cluster (green bars in Fig. 4C), is characterized by positions causing low pathogenicity, with an average value of 6.91±0.59%, suggesting that these sites are non critical for resistance. These low pathogenicity mutants are scatter along the entire 21-nt region. The second cluster contains mutants of intermediate pathogenicity (yellow bars in Figure 4C), with an average value of 36.36±4.71%, suggesting that these positions are moderately important for amiRNA-mediated resistance. Most of these medium effect mutants are located between nucleotides 10 and 18, with the exception of m2, which is located at the 3′ end of the target sequence. Finally, the third cluster contains those mutants with a greater likelihood of resistance breakdown (red bars in Figure 4C). On average, these large effect mutants have 81.85±4.14% pathogenicity, highlighting their importance in amiRNA-mediated resistance. These important sites mostly congregate on the 3′ third of the target sequence, plus m9 and m12 which are located in the center of the sequence.

In good agreement with the pathogenicity data, symptoms elicited by these mutants were generally delayed in comparison with TuMV-GFP (Table 1). For the 9 small-effect mutants, the median delay in symptom development was two days, for the medium effect mutants, two days, and for the large-effect mutants, only one day.

### Evolution of viral genomes that causes breakdown of amiR^159^-P69–mediated resistance

We sought to gain deeper insights into the question of why different substitutions within the 21-nt target sequence overcame the amiRNA-mediated resistance to different degrees. To address this issue, we recovered viral RNAs from symptomatic leaves of amiR^159^-P69 plants and analyzed the 21-nt target sequence on the viruses by RT-PCR and sequencing. Several possibilities could account for the resistance breakdown. (1) The scanning mutation could affect amiR^159^-P69-mediated cleavage to different degrees in amiR^159^-P69 plants. (2) The scanning mutant virus could undergo rapid evolution accumulating additional mutation(s) within the target site to further increase the number of mismatches and consequently the ability to replicate in presence of the amiR^159^-P69. (3) Since the 21-nt sequence and the *GFP* gene are non-essential for virus survival, the surviving mutant virus could undergo in-frame deletions in this region that would render the virus unrecognizable by the amiR^159^-P69. To discriminate amongst these possibilities, we designed two primers (PTuNIb-8671 and MTuCP-8982) to amplify an 1136 bp DNA fragment including a partial *NIb* gene, *GFP* gene, the target 21-nt sequence, and a partial *CP* gene (Fig. 1B). This primer set can be used to check for any possible deletion within the *GFP*-21nt sequence. The recovery of lower molecular mass PCR fragment(s) would indicate deletion of this region. In addition, we designed another primer set (PXFP-532 and MTuCP-8982) to amplify a 482-bp fragment that included a partial *GFP* gene and a partial *CP* gene (Fig. 1B). This 482-bp DNA fragment can be used to analyze sequences surrounding and within the 21-nt target site.

**Figure 5:**
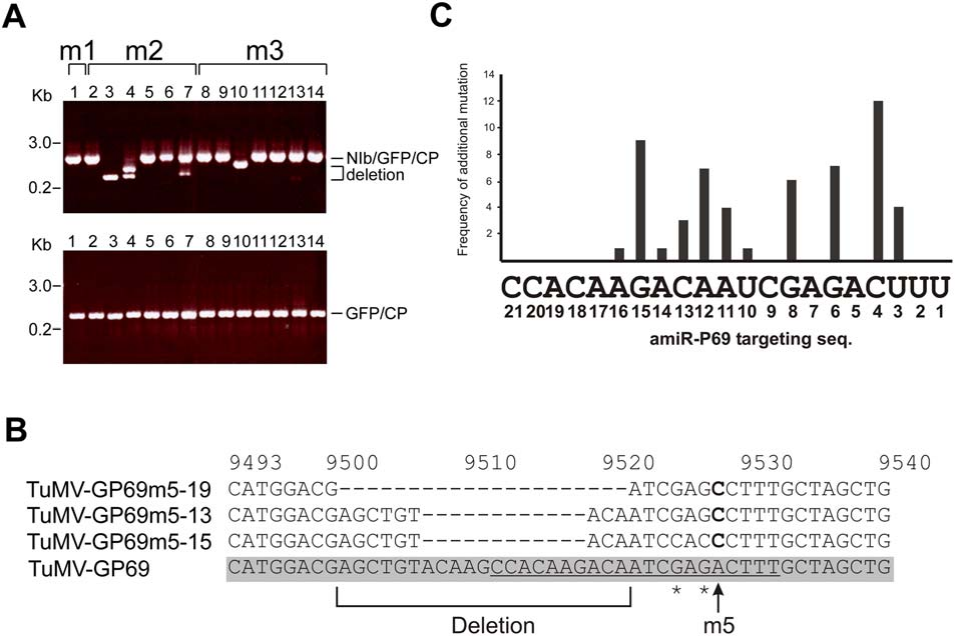
Sequence analysis of chimeric TuMV viruses recovered from susceptible amiR^159^-P69 transgenic plants. (A) Representative RT-PCR results of chimeric TuMV viruses derived from susceptible transgenic plants infected with m1, m2, and m3. The NIb-CP (top panel) and GFP-CP regions (bottom panel) of scanning mutants virus TuMV-GP69m1 (m1; lane 1), TuMV-GP69m2 (m2; lanes 2–7), and TuMV-GP69m3 (m3; lanes 8–14) were checked for deletion of the 21-nt target sequence by RT-PCR. (B) Representative results of chimeric TuMV viral sequences with deletion in the 21-nt target site. The sequence of TuMV-GP69 was used as the standard sequence (gray box), and the 21-nt target site was underlined. Representative sequences of three scanning mutant viruses, TuMV-GP69m5-13, 15, and 19, from susceptible plants were aligned. Nucleotide mutation in position 5 is in bold and indicated with an arrow. Additional mutations are marked by asterisks. (C) Frequency of additional mutation on the 21-nt target site. The *x*-axis shows the 21-nt sequence on TuMV-GP69. Numbers below indicate the positions of amiR^159^-P69 starting from the 5′ end. Bars show frequency of additional mutations in scanning mutant viruses recovered from susceptible plants.

Figure 5A top panel shows that several virus sequences recovered from symptomatic amiR^159^-P69 plants contained deletion of the *NIb*-*CP* gene, such as TuMV-GP69m2 (lanes 3, 4 and 7) and TuMV-GP69m3 (lanes 10 and 13). In addition, several viruses, such as TuMV-GP69m5-13, -15, and -19 contained partially-deleted 21-nt sequence (Fig. 5B). Moreover, TuMV-GP69m5-15 also accumulated two additional mutations on positions 6 and 8 (Fig. 5B).

We selected viruses with no deletion on the *NIb*-*CP* gene region and sequenced the *GFP*-*CP* gene regions (Figure 5A, bottom panel). Our results showed that virus sequences recovered from symptomatic amiRNA plants contained additional mutations within the 21-nt target site (Table 2). These scanning mutant viruses have 1–3 additional mutation(s) on the 21-nt target site and most of additional mutations introduced additional mismatches (Table 2). Only 11 out of the 21 positions showed additional mutations. These 11 positions are 3, 4, 6, 8, and 10–16 (Figure 5C). Positions 8, 14 and 16 were of little importance for amiRNA mediated resistance (see above), whereas all other 8 positions had either a moderate or a large effect on the likelihood of escaping the amiRNA-mediated resistance. All together, our results indicate that up to 2 mutations on critical positions within the 21-nt sequence can overcome specific resistance.

**Table 2:** Additional mutations on the 21-nt target fixed during virus evolution.

amiR^159^-P69 seq.		21	20	19	18	17	16	15	14	13	12	11	10	9	8	7	6	5	4	3	2	1
		G	G	U	G	U	U	C	U	G	U	U	A	G	C	U	C	U	G	A	A	A
		:	:	:	:	:	:	:	:	:	:	:	:	:	:	:	:	:	:	:	:	:
Targeted seq.		C	C	A	C	A	A	G	A	C	A	A	U	C	G	A	G	A	C	U	U	U
TuMV-GP69-	#																					
m2^1^	1											C					A				**C** 3	
	4							A									C		U		**C**	
	6																A				**C**	
	7											G	C								**C**	
m3	4								G											**C**		
	11							A												**C**		
	10										U									**C**		
	13														A					**C**		
	14										C									**C**		
m4	4																		**A**	C		
	6							A											**A**	C		
	11														A				**A**			
	14										U								**A**	C		
	15							A											**A**			
	16														U				**A**			
m6	1							A									**A**					
	7																**A**		U			
	5														A		**A**					
	10											C					**A**					
	13							U									**A**					
m7	1															**C**			U			
	2															**C**	A					
	13									A						**C**						
m10	2										U		**C**									
	3												**C**						U			
	4						C						**C**									
	5							A					**C**						U			
m11	1											**C**							U			
	8											**C**			A							
	9										G	**C**										
m12	3										**C**				U							
	4									U	**C**								U			
	5										**C**								U			
	17										**C**						A		U			
	18										**C**						U					
m13	2							A		**A**												
m14	5								**C**										U			
	6								**C**		G						U					
m15	1							**A**											U			
	2							**A**												C		
m17	1					**C**		A														
m18	2				**A**														U			
	4				**A**						U											
	9				**A**							G										
	10				**A**					U												
A. M. F^2^							1	9	1	3	7	4	1		6		7		12	4		

1 m2, m3, m4, …, m18 = mutant virus that single-mutated on targeted 21-nt seq.

2 A.M.F. = frequency of additional mutation.

3 The original mutation generated by PCR mutagenesis is in bold.

Interestingly, we found that 40 out of 55 observed additional mutations were transitions. Over 50% of the additional mutations in positions 3, 4, 6, 8, 11, and 15 were transition mutations. For example, there were 100% U→C or C→U transitions in position 3 and 4. In position 15, the G→A transition represented 88.89% of all observed mutations at this particular site. Moreover, there were 50 to 66.67% of G→A transitions at positions 6, 8, and 11. This result is not surprising, since it is well known that virus coding regions show an excess of transitions over transversions [20],[21]. Three reasons can account for this bias: (i) the underlying mechanisms of mutation render transitions easier than transversions, (ii) the redundancy of the genetic code is expected to make the average effect of a transition smaller than the average effect of a transversion, and (iii) RNA editing by deaminase-like enzymes have been shown to induce transition mutations in single-stranded regions of certain viral genomes [22],[23]. Our results show that transitions rather than transversions also mainly accumulate in viral sequences, such as that of the target of amiR^159^-P69, which are not under the selective constrain imposed by being a coding sequence. Furthermore, not all transitions are equally represented in Table 2, since G→A (17/40) and C→U (14/40) are significantly over represented (χ^2^ = 12.600, 3 d.f., *P* = 0.006). This bias amongst transitions is expected if the viral RNA was edited by cytidine deaminase enzymes.

## Discussion

### A 21-nt sequence is necessary and sufficient for amiRNA-specific resistance

Here, we have developed a heterologous-virus resistance system to study and identify critical positions of amiRNA target site for amiRNA-mediated resistance. The amiR^159^-P69 transgenic plant were resistant to TYMV, but not to TuMV (a heterologous-virus), because there was no sequence homology with amiR^159^-P69 on the TuMV viral genome [4]. However, the chimeric heterologous-virus TuMV-GP69 carrying the 21-nt sequence of *P69* gene cannot infect amiR^159^-P69 plants because of amiR^159^-P69-mediated cleavage. By contrast, the TuMV-GP69m virus with mutations on the central region within this sequence is sufficient to prevent amiRNA-mediated cleavage on the viral RNA and compromise specific virus resistance. These results indicated that the 21-nt target site is portable and is necessary and sufficient to confer virus resistance.

Because of the genome organization and proteolytic processing strategy of potyvirus, TuMV can express GFP when a cDNA for this protein is inserted in-frame between the *NIb* and *CP* genes. The encoded GFP protein contains two NIa proteinase cleavage sites (CVYHQ/A) at the N- and the C-terminus such that GFP can be released from the viral polyprotein by proteolytic processing. In addition, the additional 21-nt target site that encodes seven amino acids is also nonessential for TuMV. Therefore, any modification on the GFP gene and the 21-nt target site would not affect the chimeric TuMV as evidenced by its ability to infect plants and stably replicate.

### Critical position for amiRNA-mediated resistance

Using an *in vivo* assay we identified critical positions on the 21-nt target sequence for RISC-amiRNA-mediated cleavage. Scanning mutations on the 21-nt target site of the challenging chimeric virus showed different degree of resistance breakdown on amiR^159^-P69 transgenic plants. Based on the proportion of amiR^159^-P69 plants that become susceptible, we defined critical, moderately critical and non-critical positions on the 21-nt sequence. Positions 3–6, 9, and 12, are found to be critical for resistance because chimeric virus with mutations at these sites were pathogenic, on average, on ∼82% of amiR^159^-P69 plants. Positions 2, 10, 11, 13, 15, and 18 are classified to be moderately critical mutations giving average pathogenicity of ∼36% in transgenic plants. The remaining positions are classified as non-critical for resistance since mutants at these sites were only pathogenic in less than 7% of inoculated plants. In summary, most critical positions are localized on sequences complementary to the 5′ portion of the amiRNA whereas the moderate critical positions are mainly localized on the central region of the target site. The exception being position 18, which is complementary to the 18^th^ nucleotide on 3′ portion of the amiRNA, and was also moderately important for amiRNA-mediated resistance. These results are consistent with those obtained with *in vitro* miRNA-mediated cleavage using a wheat germ system [16]. All together, results suggest that the 5′ portion of the miRNA is more important in governing the specificity of miR165/166 regulation [16]. Furthermore, the “two-state model” for RISC-mediated target recognition also proposes that the specific interaction between RISC and the substrate is initiated via the 5′ portion of siRNA, as the 3′ portion is less favorably structured to undergo base pairing before the initial recognition of a mRNA target [17].

### Possible mode of breakdown induced by critical-position mutation

**Figure 6:**
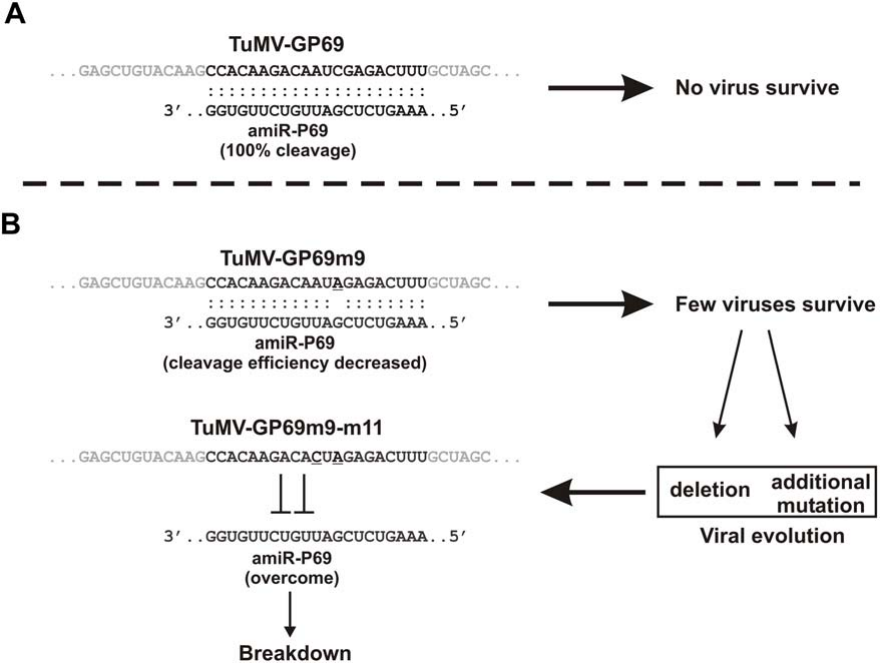
A working model to explain breakdown of amiRNA-mediated resistance by virus mutation. (A) Complete sequence complementarity between the 21-nt target site and the amiRNA. (B) TuMV-GP69m9 is a mutant virus with a single mutation (underlined) on position 9 of the target site. As this position is critical, the mutation causes a decrease in the cleavage efficiency of TuMV-GP69m9 viral RNAs, allowing some viral RNAs to escape the amiRNA-mediated surveillance. The surviving TuMV-GP69m9 virus rapidly undergoes evolution, collecting additional mutations on the target site. The next generation of mutated viruses with additional mutations can overcome the amiRNA-mediated resistance.

Representative results showed that several virus sequences recovered from symptomatic amiRNA plants contained deletions and additional mutations within the 21-nt target site. This observation is consistent with the hypothesis that mutations in certain critical positions within the target site reduced amiRNA-mediated cleavage efficiency (Figure 6A). The reduced RISC activity allowed certain mutant viruses to escape amiRNA-mediated cleavage, allowing them to replicate and complete an infectious cycle (Figure 6B). During subsequent virus replication, additional mutations or deletions of the target sequence would be positively selected because they would escape from the amiRNA-mediated specific resistance more efficiently (Figure 6B). Indeed, the effect of miRNA-mediated cleavage was drastically alleviated in transgenic plants expressing the silencing suppressor P1/HC-Pro. Chimeric *Plum pox virus* (PPV) carrying an endogenous miRNA target site can also overcome the resistance by deletion and mutation on the 21-nt target sequence [24]. Finally, deletions, in addition to point mutations, are also a very common pathway taken by HIV-1 to escape from RNAi-based therapy in cell culture experiments [10],[13]. In general, these deletions have a major impact on the local RNA secondary structure, creating new hairpin structures not accessible to the siRNAs [13].

In some deletion mutant viruses recovered from breakdown plants, the entire *GFP* gene, along with small portions of the *NIb* gene C-terminus or the *CP* gene N-terminus, has been deleted from the viral genome (data not shown). These results suggest that TuMV can tolerate small deletions in the *NIb* or *CP* gene. In addition, deletions in between the *GFP* gene and the target site (Fig. 5B) may be triggered by polymerase-jumps on repeat sequence (ACAA).

Widespread plant miRNA-directed translational repression as an important miRNA-mediated regulatory mechanism in plants has recently been reported [25]. Imperfect pairing with central mismatches in small RNA-target hybrids promotes translational repression because it excludes slicing [25]. This observation suggests the possibility that imperfect pairing between the amiRNA and mutant target sequences might lead to translational repression rather than viral RNA cleavage. In contrast to the catalytic effects of amiRNA-mediated viral RNA cleavage, translational repression requires stoichiometric amounts of amiRNAs and therefore is not as efficient. Inefficient translation inhibition might allow residual virus replication and progeny virus can still escape the repression by fixing changes in the target sequence.

In this study, we have provided evidence that the 21-nt target site is necessary and sufficient for amiRNA specific resistance and we have also identified several positions on the target site that are critical for this resistance. These results are clearly important for future design of amiRNA-mediated virus resistance. Highly conserved regions on viral genomes should be selected as target sites to minimize the likelihood of fixation of mutations responsible for resistance breakdown, because these mutations might affect viral protein function and hence have a negative impact on virus fitness and survival. Furthermore, several amiRNAs targeting different conserved regions on a viral genome could be co-expressed in transgenic plants to minimize the chances of resistance breakdown. Finally, the heterologous-viral system described here also can be used for viral evolution studies in the future.

### Patterns of molecular evolution in the amiR^159^-P69 target

As we have highlighted several times here, the amiR^159^-P69 target sequence inserted in the TuMV-GFP genome is functionally neutral. This has allowed us to separate selective pressures acting on the protein level from those acting on the RNA level. Consequently, the patterns of molecular evolution should be different. Not surprisingly, and in agreement with previous data obtained with other viruses, we have observed that most of the mutations fixed within the target were transition mutations [21]. We consider it striking that 77.50% of these fixed transitions were of the type G→A and C→U. These transitions are from the particular type induced by cellular cytidine deaminases (CDAs) involved in innate immune responses to viral infection [26], a phenomenon particularly well described for HIV-1 and other retroviruses [23],[27] but never before on an RNA virus. This observation prompted us to hypothesize that as an antiviral strategy plants may have an RNA-editing system that induces hypermutagenesis in viral genomes. *A thaliana* contains a family of nine paralogous genes that are annotated as CDAs owing to their homology to *CDA1* [28]. These nine genes are good candidates to explore whether their gene products possess cytidine deaminase activity and whether they are indeed involved in plant antiviral defense.

## Materials and Methods

### Plant material and growth conditions

Two amiRNA transgenic *A. thaliana* lines, amiR^159^-P69 and amiR^159^-HC-Pro, were used in this study [4]. Plants of *N. benthamiana* were transformed with *Agrobacterium tumefaciens* containing the pBA-amiR^159^-P69 plasmid by standard methods. T2 transgenic *N. benthamiana* (a mixture of homozygotes and hemizygotes) were analyzed for transgene and miRNA levels and 4 independent lines (#1, 2, 3, and 4) with high amiR^159^-P69 expression levels were selected for virus challenge experiments. Seeds were surface-sterilized and chilled at 4°C for 2 d before being placed on Murashige and Skoog (MS) medium with/without antibiotics or sowed directly on Florobella potting compost/sand mix (3∶1). Plants were maintained in a growth room (16 h light/8 h darkness, 20 to 25°C).

### Northern blot hybridizations

Ten µg of total RNA was resolved in a 15% polyacryamide/1× TBE (8.9 mM Tris, 8.9 mM boric acid, 20 mM EDTA)/8M urea gel and blotted to a Hybond-N+ membrane (Amersham). DNA oligonucleotides with the exact reverse-complementary sequence to miRNAs were end-labeled with ^32^P-γ-ATP and T4 polynucleotide kinase (New England Biolabs) to generate high specific activity probes. Hybridization was carried out using the ULTRAHyb-Oligo solution according to the manufacturer's directions (Ambion) and signals were detected by autoradiography. In each case, the probe contained the exact antisense sequence of the expected miRNA to be detected.

### Construction of infectious clones of TuMV-GP69 and TuMV-GP69m chimeric viruses

The TuMV infectious clone (p35STuMV-GFP) comprises of a 35S promoter and the full-length cDNA of TuMV-GFP. The *GFP* gene was inserted between *NIb* and *CP* genes. This chimeric TuMV-GFP virus was used as a surrogate wild type virus and as a backbone for construction of various chimeric recombinant viruses described here.

We fused the 21-nt sequence (5′-CCACAAGACAAUCGAGACUUU-3′) of the TYMV P69 gene targeted by amiR^159^-P69 to the 3′ end of the *GFP* gene. The GFP-P69 fusion sequence was then inserted in between the *NIb* and *CP* genes to generate the p35STuMV-GP69 infectious clone. As a control, the central 4 nts (underlined) of the 21-nt target sequence (5′-CCACAAGACCUGAGAGACUUU-3′) was mutated to give GFP-P69m which was also inserted in the same position of the virus to generate p35STuMV-GP69m.

### Scanning single nucleotide mutagenesis on the amiR^159^-P69 target site

As the 21-nt target sequence is in a non-essential region of the TuMV-GP69 it can be altered without affecting essential viral gene function. We performed serial single nt mutagenesis from the 1^st^-nt to the 21^st^-nt of the target site on the TuMV-GP69 infectious clone by PCR mutagenesis and the resulting series of scanning mutants were confirmed by sequencing. A total of 21 single-nt substitution recombinant viruses were generated. Based on the mutation position, the recombinant viruses were named TuMV-GP69mX, in which X refers to the mutation position. For example, the mutant with substitution on the 1^st^-nt of the target site was named TuMV-GP69m1.

### Protocol of challenge inoculation with recombinant viruses and pathogenicity estimation

To evaluate the efficiency of amiRNA-mediated specific resistance toward wild type and mutant viruses, we have established a standard protocol for virus challenge inoculation and quantitative evaluation of pathogenicity. Our overall aim was to reduce the time for virus maintenance and propagation in host plants so as to minimize possible virus evolution. All recombinant viruses were propagated from DNA infectious clones. Aliquots of 20 µL, containing 1 µg of DNA in sterilized water, were mechanically applied onto carborundum-dusted leaves of *Chenopodium quinoa* Willd with a sterilized glass spatula. Seven days post-inoculation (dpi), local lesions were obtained on inoculated leaves. Viruses were then isolated from single lesions and transferred to *N. benthamiana* for amplification. Four dpi leaves of *N. benthamiana* with viral infection symptoms were used as the source of inoculum to challenge WT and amiR^159^-P69 *N. benthamiana* plants for evaluation of virus pathogenicity (i.e., frequency of break-down). Twenty amiR^159^-P69 plants were used for each experiment, and the experiments were repeated 3 times. Resistance efficiency of amiR^159^-P69 plants challenged with recombinant viruses were compared with those obtained with control viruses, including TuMV-GFP, TuMV-GP69 and TuMV-GP69m.

Pathogenicity was evaluated between two and four times for each one of the 21 TuMV-GP69mX recombinant viruses. Count data from experiments that were statistically homogeneous were pooled into a single set, whereas experiments that behaved as outliers were removed from the dataset.

### Sequence analysis of the 21-nt target region

In plants displaying symptoms, it was important for us to verify the sequence of the 21-nt target site to ensure that no other mutations had occurred to confound our results. To this end, total RNA was extracted from infected leaf tissues using the Trizol reagent (Invitrogen). One µg total RNA was used for reverse-transcriptional polymerase-chain reaction (RT-PCR) with PTuNIb-8671 (5′-GAACCAGCTCAAGAGGATCT-3′) and MTuCP-8982 (5′-GCCACTCTCTGCTCGTATCTTGGCACGCGC-3′) for amplification of the viral region between the partial *NIb* and the *CP* genes (Fig. 1B). The PCR fragments then were analyzed by sequencing.

### Statistical analyses

The pathogenicity of different recombinant viruses was estimated as the frequency of infected plants out of the total number of inoculated plants. The LaPlace's point estimator for the Binomial frequency parameter was used instead of the commonly used maximum likelihood estimator [29]. The LaPlace method provides a more robust estimate of the Binomial parameter for small sample sizes [30]. Binomial 95% confidence intervals (CI) were also computed.

TuMV-GP69mX recombinant viruses were classified into groups of similar pathogenicity using the two-step cluster analysis [31]. In brief, this method classifies data in groups that minimize the within-group variance whilst maximizing the among-groups variance. The method starts with the simplest model (i.e., all viruses are equally pathogenic) and computes its likelihood; then, it classifies the mutants into two clusters and computes the likelihood of this model; finally, it does the same for three clusters, four clusters and up to 21 clusters (i.e., each site behaves in a different way and no classification is possible). For each model, Schwarz's Bayesian information criterion (*BIC*) was used as a measure of the goodness-of-fit and the model with the lowest *BIC* was considered to be the best one [32].
